# Sintering-Resistant Nanoparticles in Wide-Mouthed Compartments for Sustained Catalytic Performance

**DOI:** 10.1038/srep41773

**Published:** 2017-02-03

**Authors:** Jia Liu, Qingmin Ji, Tsubasa Imai, Katsuhiko Ariga, Hideki Abe

**Affiliations:** 1 World Premier International (WPI) Research Center for Materials Nanoarchitectonics (MANA), National Institute for Materials Science (NIMS), Namiki 1-1, Tsukuba, Ibaraki 305-0044, Japan; 2 Environment and Energy Materials Division, National Institute for Materials Science (NIMS), Namiki 1-1, Tsukuba, Ibaraki 305-0044, Japan

## Abstract

Particle sintering is one of the most significant impediments to functional nanoparticles in many valuable applications especially catalysis. Herein, we report that sintering-resistant nanoparticle systems can be realized through a simple materials-design which maximizes the particle-to-particle traveling distance of neighbouring nanoparticles. As a demonstration, Pt nanoparticles were placed apart from each other in wide-mouthed compartments tailored on the surface of self-assembled silica nanosheets. These Pt nanoparticles retained their particle size after calcination at elevated temperatures because the compartment wall elongates the particle-to-particle traveling distance to preclude the possibility of sintering. Moreover, these Pt nanoparticles in wide-mouthed compartments were fully accessible to the environment and exhibited much higher catalytic activity for CO oxidation than the nanoparticles confined in the nanochannels of mesoporous silica. The proposed materials-design strategy is applicable not only to industrial catalysts operating in harsh conditions, but also opens up possibilities in developing advanced nanoparticle-based materials with sustained performance.

Dispersed nanoparticles (NPs) are of universal importance in the environment, energy-conversion technologies and biomedical applications. Particle sintering represents the general mechanism leading to destabilization of such dispersed NPs through particle migration-coalescence and/or Ostwald ripening^1^,^2^,^3^,^4^,^5^,^6^. Indeed, the performance of advanced functional nanoparticles such as photo-sensitivity of plasmonic NPs or light-emission performance of luminescent NPs is frequently degraded by particle sintering^7^,^8^,^9^,^10^. In particular, particle sintering is one of the most critical issues for catalytic NPs operating in harsh conditions including elevated temperatures, overpotentials or light illumination^1^,^5^. To mitigate particle sintering, strategies have been developed focusing on steric protection/confinement of nanoparticles by coated with porous shells^11^,^12^,^13^,^14^,^15^, sandwiched between solid cores and porous shells^16^,^17^,^18^,^19^,^20^, or encapsulated in the nanometer-sized channels of zeolites and their silica- and metal-organic framework mimics^21^,^22^,^23^,^24^. However, such confined structures passivate surface active sites of the NPs and are often accompanied by decrease in mass- and/or energy transfer, resulting in diminished catalytic activity. Laying emphasis on enlarging the particle-to-particle traveling distance of neighboring NPs through a rational design of the support materials, we here report a simple yet general strategy to prevent functional NPs from unfavorable sintering without sacrificing their intrinsic performance.

As illustrated in Fig. 1a, NPs dispersed on the open surface of support materials can migrate all over the two-dimensional surface through Brownian motion and/or atomic diffusion. The neighboring two NPs with a particle-to-particle traveling distance, *d*, readily encounter to each other and grow into large particles when *d* < 2*r*, where *r* corresponds to the migration length of individual NPs at a given temperature. The NPs encapsulated in porous supports (Fig. 1b) are limited in mobility along the one-dimensional nanochannels, but the NPs residing in the same channel are still ready to grow into large particles when *d* < 2*r*. As distinct from these typical forms of supported NPs, here suppose a material having a number of compartments with wide-mouth openings on the surface (Fig. 1c). When the individual compartments are occupied by a single NP, the particle-to-particle traveling distance *d* is much larger than the spacing of neighboring particles due to the presence of the compartment wall. Even though the NPs and/or their constituent atoms can freely migrate over the compartment surface, the possibility of sintering is greatly lowered on account of the overlong traveling distance (*d* > 2*r*)^24^,^25^.

## Results

**Figure 1:**
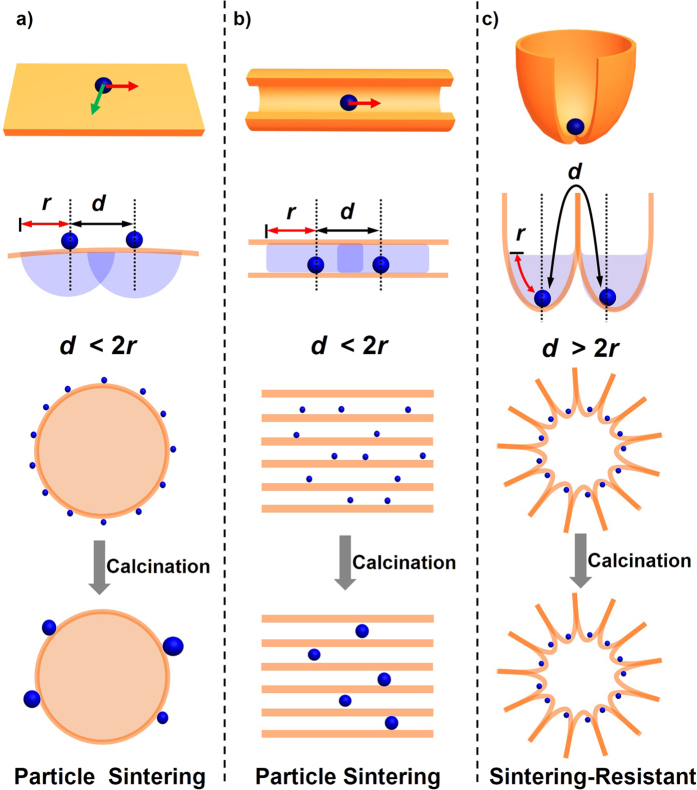
Schematic illustration of the relationships between support topology and particle sintering. Nanoparticles dispersed on (**a**) the solid supports with two-dimensional open surface, (**b**) the porous supports with one-dimensional nanochannels, and (**c**) the supports enriched with wide-mouthed compartments on the surface. *d* stands for the particle-to-particle traveling distance of neighboring two particles, while *r* denotes the migration length of individual particles at a given temperature.

**Figure 2:**
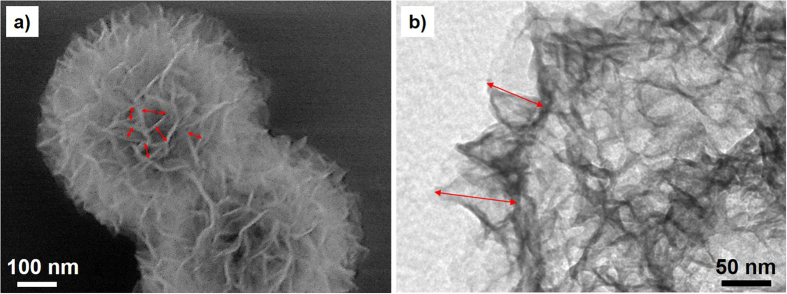
Topology/nanostructure characterization of the compartment-rich support (CMPT). (**a**) SEM- and (**b**) TEM images of CMPT. The red arrows in (**a**) and (**b**) respectively denote the opening width (10–80 nm) and depth (60–80 nm) of the compartments on the surface of CMPT.

A proof-of-concept demonstration was implemented by utilizing dendrimer-encapsulated Pt NPs (PtDEN) as the active component (Supplementary Fig. S1)^22^,^26^, and three kinds of silica supports with different topology/nanostructures (the demonstration procedure is illustrated in Supplementary Scheme S1). The compartment-rich support (CMPT) was consisted of three-dimensionally assembled silica nanosheets which afforded a large number of compartments (60–80 nm deep) with the top ends widely open (10–80 nm) (corresponding to Figs 1c and 2 and Supplementary Fig. S2)^27^. The SBA15 support, a typical mesoporous silica material which has been intensively used to encapsulate catalytic NPs^22^,^23^,^24^, had straight nanochannels with a pore size of 8.1 nm (Fig. 1b; Supplementary Fig. S3). Silica nanosphere support (NS) comprised filled silica particles with smooth surfaces (Fig. 1a; Supplementary Fig. S4, see Supplementary Table S1 for more details about the three supports).

**Figure 3:**
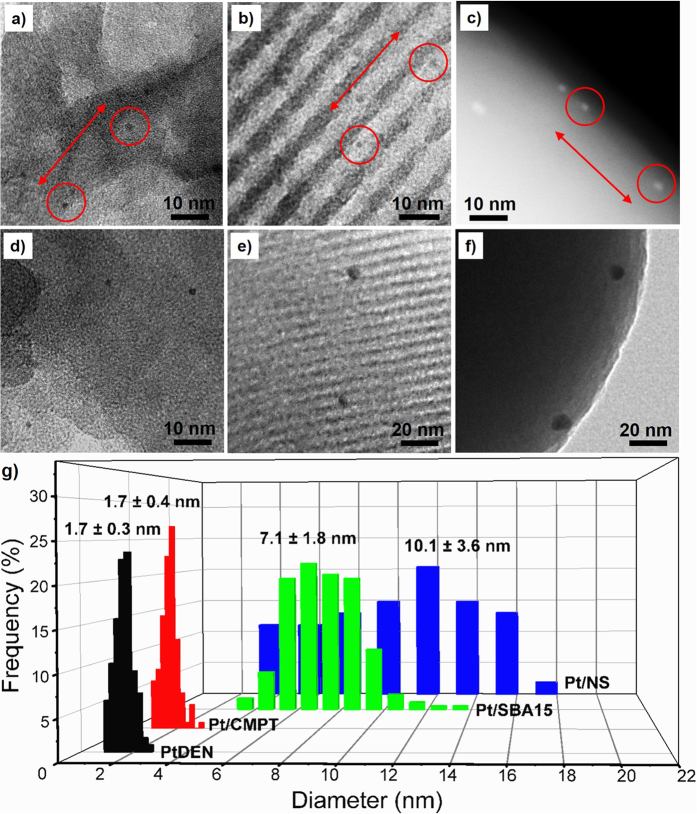
Comparison of the thermal stability of Pt particles immobilized on three supports with different topology/nanostructures. (**a**,**b**) TEM- and (**c**) HAADF-STEM images of the as-synthesized (**a**) PtDEN/CMPT, (**b**) PtDEN/SBA15 and (**c**) PtDEN/NS. TEM images of the thermally treated (**d**) Pt/CMPT, (**e**) Pt/SBA15 and (**f**) Pt/NS with identical Pt loading, and (**g**) their Pt particle size distribution compared with PtDEN. The thermal treatment was carried out by calcination in air at 550 °C for 4 h.

PtDEN (Pt particle size = 1.7 ± 0.3 nm) was first dispersed and immobilized on each of the silica supports at the same Pt loading weight (~0.07 wt%, Supplementary Table S2). There was negligible difference in Pt particle size for the obtained products, PtDEN/CMPT, PtDEN/SBA15 and PtDEN/NS (Fig. 3a,b and c; Supplementary Fig. S5). The spacing between the neighboring Pt particles (labelled in red circles in Fig. 3a,b and c) was about 30 nm. The ratio of CMPT compartments to Pt NPs (compartment/particle ratio) was calculated as larger than 7.1 (Supplementary Table S2), suggesting that each of the compartments of CMPT contained less than one Pt NP. Thus the particle-to-particle traveling distance for PtDEN/CMPT was likely much larger than 30 nm considering that the individual PtDENs were separated by the compartment walls.

These supported PtDENs were calcinated in air at 550 °C for 4 h to eliminate the dendrimer components and expose the active surface of Pt NPs^28^,^29^, yielding Pt/CMPT, Pt/SBA15 and Pt/NS (Fig. 3d,e and f). Through the calcination process, the nanostructure of CMPT was well preserved (Supplementary Fig. S6a). The high-angle annular dark field scanning transmission electron microscopy (HAADF-STEM) image of Pt/CMPT clearly shows that the Pt NPs remained small and finely separated from each other by a number of silica nanosheets (Supplementary Fig. S6b). The transmission electron microscopy (TEM) images revealed that the Pt particle size in Pt/CMPT was at 1.7 ± 0.4 nm, nearly unaffected by the high-temperature calcination, and no large Pt aggregates were observed throughout the support particles (Fig. 3d,g and Supplementary Fig. S6c). In marked contrast, after the same treatment, the Pt particle size in Pt/SBA15 and Pt/NS increased up to 7.1 ± 1.8 nm and 10.1 ± 3.6 nm, respectively (Fig. 3e–g). The elemental mapping analysis further manifested that Pt was uniformly distributed over the support particles as for Pt/CMPT, but sintered into large aggregates as for Pt/NS (Supplementary Fig. S7). High-resolution TEM indicates that all of the Pt NPs in the three samples had a face-center-cubic (FCC) structure (Supplementary Fig. S8). No atomic fringes were observed for the supports (*i*.*e*., CMPT, SBA15 or NS), indicating that the support materials had an amorphous structure (Supplementary Fig. S8).

When Pt loading of PtDEN/CMPT increased up to 0.21 wt% (compartment/particle ratio >2.5), the particle size of Pt NPs still remained as 1.7 ± 0.4 nm after calcination to obtain Pt/CMPT, indicating that most of the Pt NPs were still highly separated by the compartment walls in this situation (Supplementary Figs S9 and S10). Further raising the Pt loading to 0.35 wt%, 0.49 wt% and 0.84 wt%, the Pt NPs in Pt/CMPT grew up to 1.9 ± 1.0 nm, 2.0 ± 1.1 nm and 2.7 ± 2.1 nm, respectively, after the same calcination procedure (Supplementary Figs S9 and S10). These results support the presumption that the chance for one compartment to hold more than one Pt NP increases with decreasing the compartment/particle ratio. The Pt NPs residing in the same compartment can grow into larger particles because of the short particle-to-particle traveling distance. It should be noted that CMPT achieved good spatial separation of Pt NPs even at higher metal loading (>0.21 wt%) in view of the much smaller Pt particle size for all the CMPT-based samples than that for Pt/SBA15 and Pt/NS containing 0.07 wt% of Pt.

**Figure 4:**
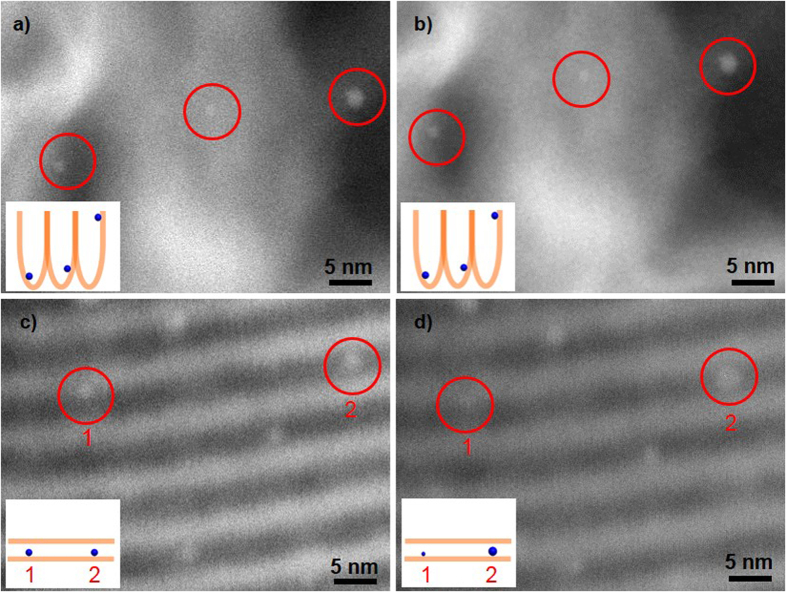
*In situ* monitoring the behaviour of Pt particles immobilized on different nanostructured supports at high temperature. HAADF-STEM images recorded *in situ* of the same area of the (**a**,**b**) PtDEN/CMPT and (**c**,**d**) PtDEN/SBA15 samples (**a**,**c**) before and (**b**,**d**) after the heat treatment at 550 °C for 4 h under high vacuum. (Inset: schematic illustration of the possible states of the Pt NPs labelled in red circles).

*In situ* heating HAADF-STEM observation was performed in vacuum at 550 °C over PtDEN/CMPT and PtDEN/SBA15 by directly heating the sample in the microscope column with a special sample holder. The results as shown in Fig. 4a and b directly indicate that the Pt NPs reside in different compartments resembling a “ball in a cup” model, rather than simply wedged in between the silica nanosheets. More importantly, neither the size nor distribution of Pt NPs (labelled in red circles in Fig. 4a and b) over CMPT was changed before and after being heated for 4 h. However, as regards the neighboring two Pt NPs (labelled in red circles, denoted as Particle 1 and Particle 2 in Fig. 4c and d) situated in the same nanochannel of SBA15, Particle 1 diminished in size whereas Particle 2 gained a larger size after heated for 4 h, suggesting the occurrence of particle sintering via an Ostwald ripening mechanism in this scenario. Though the real situation of calcination in air can be different from that in vacuum^6^, the above results support that the CMPT can indeed inhibit the unfavorable particle sintering because the particle-to-particle traveling distance exceeds twice of the atomic-diffusion length of individual NPs (*d* > 2*r*).

**Figure 5:**
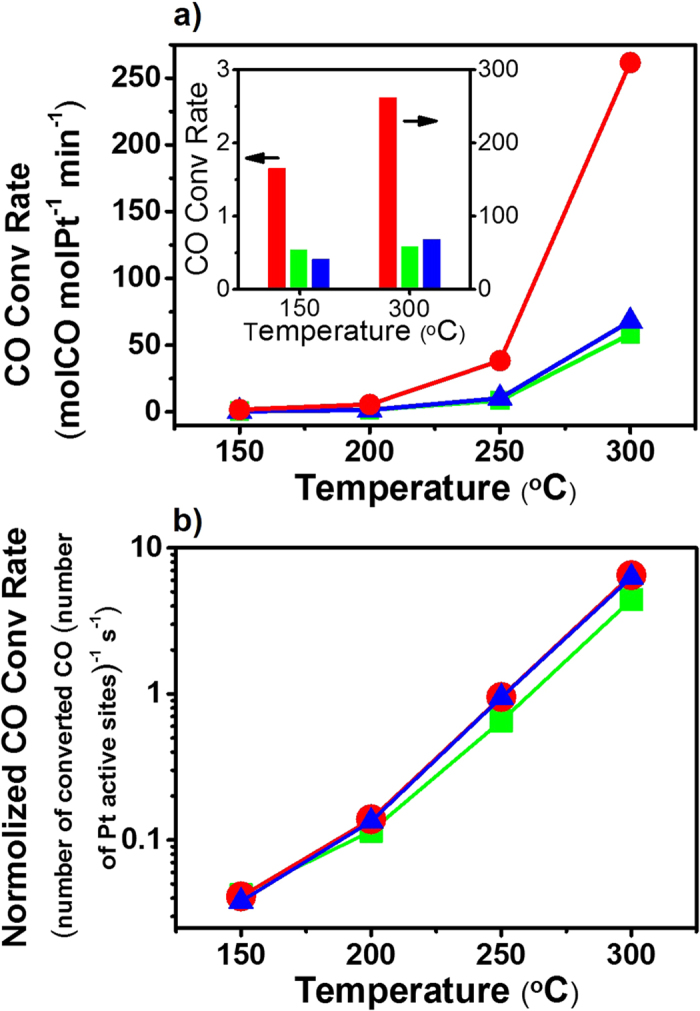
Comparison of catalytic performance in CO oxidation reaction. (**a**) CO conversion rate and (**b**) normalized CO conversion rate with respect to the number of Pt active sites, as a function of temperature for Pt/CMPT (red), Pt/SBA15 (green), and Pt/NS (blue). (Inset: comparison of the results at 150 and 300 °C in the form of bar chart).

Finally we examined the performance of the different supported catalysts (Pt/CMPT, Pt/SBA15 and Pt/NS) toward the oxidation of CO with O_2_ in a temperature range of 150–300 °C. As presented in Fig. 5a, the activity of the three catalysts all increased with increase in the reaction temperature. Significantly high CO conversion rate was observed for Pt/CMPT in the whole temperature range in comparison with Pt/SBA15 and Pt/NS. After exposure to the reactive gas atmosphere at high temperatures, the Pt NPs in Pt/CMPT remained well separated and retained their particle size, while the textural parameters of Pt/CMPT were nearly unchanged (Supplementary Table S1, Figs S11 and S12). Though the Pt particle size of Pt/SBA15 (7.1 ± 1.8 nm) was smaller than that of Pt/NS (10.1 ± 3.6 nm), the conversion rate afforded by the former was only marginally higher than the later at 150 and 200 °C, and became lower when temperature ascended to 250 and 300 °C (see the inset of Fig. 5a). Taking into account the pore size of SBA15 (8.1 nm), a portion of the nanochannels of SBA15 were likely blocked or nearly clogged by the Pt NPs. In fact, during the CO oxidation catalysis, the Pt NPs in Pt/SBA15 continuously grew (up to 7.4 ± 2.1 nm when the catalysis experiment completed) accompanied by a reduction in BET surface area of the sample (Supplementary Table S1, Figs S11 and S12), suggesting that the pore-blocking of Pt/SBA became more serious as CO oxidation proceeded and the reaction temperature increased. The eventual mass transfer resistance probably accounted for the lower catalytic activities of Pt/SBA15 at higher temperatures, when compared with Pt/NS which also showed a slightly increased Pt particle size (12.1 ± 3.5 nm) when completing the catalysis experiment yet facilitated fast diffusion of reactants and products over the open surface.

Figure 5b displays the normalized CO conversion rates with respect to the number of Pt active sites, namely the number of molecules of CO converted per active site of Pt particles per second, as a function of temperature for the three catalysts. CO chemisorption measurement was adopted to evaluate the numbers of metal active sites (see Methods section). The normalized CO conversion rate defined herein could be seen as a multiplication of TOF and effectiveness factor (η). Pt/CMPT and Pt/NS exhibited virtually the same values at any measured temperature in Fig. 5b. This indicates that the CMPT support provided the reactants with a fully-open access to the catalytic Pt NPs, the same as that of the NS support (η close to unity 1). Pt/SBA15 exhibited lower normalized CO conversion rates than the other two catalysts especially at high temperatures, which substantiates that SBA15-based supports are subject to constraints on mass transfer due to the pore-blocking by Pt NPs (η below unity 1). These results demonstrate the significance of the compartmented supports which not only enable the creation of small, sintering-resistant NPs with a great number of catalytic sites on the surface, but also allow reactant molecules to openly access to these catalytic sites with minimized diffusion limitation.

## Discussion

In this paper, we report a strategy to materialize sintering-resistant and open-access functional-NPs systems. Unlike the conventional methods confining functional NPs in protection materials, which most often lead to passivated active sites and poor mass- or energy transport, our emphasis is placed on elongation of the particle-to-particle traveling distance by utilizing support materials with large numbers of wide-mouthed compartments on the surface. Aside from silica, this proposed strategy is applicable to other reported three-dimensional nanosheet-assemblies including a wide range of oxides, such as Al_2_O_3_, TiO_2_, CeO_2_, Fe_2_O_3_, NiO, ZnO, MnO_2_, Co_3_O_4_, V_2_O_5_, WO_3_, as well as the manifold nanostructure arrays^30^,^31^,^32^,^33^,^34^,^35^,^36^,^37^,^38^,^39^,^40^. For example, compartmented NiO or ZnO loaded with monodispersed Cu NPs (acting as promoters) are promising for durable methane reforming^41^,^42^; Pt NPs on compartmented WO_3_ or TiO_2_ can serve as illumination-tolerant photocatalysts; compartmented graphene nanosheets would be efficient fuel-cell catalysts when combined with Pt NPs. As demonstrated here, the proposed strategy can be extended to a vast number of combinations of NPs and support materials. The materials design of unprotected and sintering-resistant NPs in open-access compartments will prompt the development of advanced functional nanomaterials with enhanced and sustained performance in practical applications.

## Methods

### Preparation of Pt dendrimer-encapsulated nanoparticles

Pt dendrimer-encapsulated nanoparticles (PtDEN) were prepared via literature method^12^. Firstly, a dendrimer stock solution (250 μM) was prepared by adding calculated amount of water to the G6-OH methanol solution. An aqueous solution of K_2_PtCl_4_ (10 mM, 5 mL) was then mixed with the dendrimer stock solution in a round-bottomed flask to obtain the desired Pt: G6-OH molar ratio of 40:1. The flask was purged with N_2_ for 30 min, sealed tightly with a septum and stirred at room temperature for 66 h to achieve complete complexation of Pt^2+^ with the tertiary amines within the dendrimer interior. These precursor complexes were then reduced using a 20-fold molar excess of NaBH_4_ from a freshly prepared aqueous solution (0.5 M) and the reduction was allowed to proceed for 8 h. Afterwards the reaction solution was purified by dialysis against 2 L of water in cellulose dialysis sacks with a molecular weight cutoff of 12000 (Sigma-Aldrich). The dialysis process occurred over 48 h with the water changed 4 times.

### Preparation of silica supports with different nanostructures

Silica nanospheres (NS) with average diameter of 500 ± 20 nm were commercial available from Nissan Kagaku Co. (Japan). The compartment-rich silica (CMPT) was synthesized according to the previously reported method with a little modification (the reactant amounts were all reduced by half relative to the reported recipe)^27^. Briefly, NS were collected from silica colloid solution by centrifugation and subsequently calcinated in air at 550 °C for 6 h. The obtained white powder (120 mg) was dispersed in water (5 mL) by sonication for 1 h. NaBH_4_ (0.5 g) was added to the above solution under vigorous stirring and the mixture was soon transformed into a 20 mL Teflon-lined steel autoclave and incubated at 75 °C for 20 h. The resulting sample was collected by centrifugation, washed several times with water until the pH of washings became neutral, and then freeze-dried. Mesoporous silica SBA15 was synthesized using the conventional method^43^. In a typical preparation, P123 (0.4 g) was dissolved in water (3 g) and hydrochloric acid solution (2 M, 12 g) with stirring at 35 °C for 4 h. Then TEOS (0.85 g) was added into the solution and the resulting mixture was stirred at 35 °C for 20 h and aged at 100 °C for an additional 24 h. The white solid product was recovered by centrifugation, washed several times with water and ethanol, and freeze dried. Thereafter, the as-synthesized sample was calcinated in air at 550 °C for 6 h to remove the template molecules.

### Preparation of silica-supported Pt nanoparticle catalysts

PtDEN stock solution was diluted with water to get a final concentration of 0.07 mg mL^−1^. CMPT or NS powders (50 mg) were dispersed in water (5 mL) by sonication for 1 h to get a uniform dispersion. Then the desired volume (0.5 mL–6 mL, theoretically corresponding to 0.07–0.84 wt% Pt loading) of PtDEN dilute solution was added and the obtained mixture was shaken at 1200 rpm overnight. As for SBA15, to enable a homogeneous dispersion of PtDEN within the silica nanochannels, the silica powder (50 mg) was dispersed in water (2.5 mL) and ethanol (2.5 mL) by sonication for 1 h, followed by the addition of PtDEN dilute solution, and then the obtained mixture was sonicated for 4 h at room temperature^44^. In both cases the precipitates were separated by centrifugation, washed several times with water, and freeze-dried. The prepared samples were denoted as PtDEN/CMPT, PtDEN/SBA15, and PtDEN/NS, respectively. The thermal treatment of these as-synthesized samples was carried out by calcination in air at 550 °C for 4 h, which simultaneously removed the organic dendrimer molecules. The finally yielded samples were represented as Pt/CMPT, Pt/SBA15 and Pt/NS, respectively. Unless otherwise specified, the Pt loading for the above samples was controlled close to 0.07 wt%.

### CO oxidation catalysis

CO oxidation measurements were performed in a circulating-gas reactor under excess O_2_ conditions in the temperature range of 150–300 °C. The thermally activated catalysts Pt/CMPT, Pt/SBA15 and Pt/NS were reduced in flowing H_2_ (5%) at 200 °C for 1 h, and vacuum-dried in the reactor at 150 °C prior to the catalytic test. A gas mixture of CO (8.47 kPa) and O_2_ (15.63 kPa) was circulated through the catalyst (25 mg) at a given temperature in the reaction line by using a recirculation pump. The volume of the circulation tube is 133.9 mL. Gas chromatograph (Shimadzu GC-8A) was used to separate the products for analysis. The reaction time for measuring CO conversion rate was in the range of 3–35 min. Normalized CO conversion rate was calculated by dividing CO conversion rate by the amount of available Pt surface active sites determined by CO chemisorption, according to which Pt/CMPT, Pt/SBA15 and Pt/NS exhibited a metal dispersion value of 67%, 22%, and 18%, respectively.

### Characterization

Transmission electron microscopy (TEM), high-resolution transmission electron microscopy (HRTEM) images, high-angle annular dark field scanning transmission electron microscopy (HAADF-STEM) and energy dispersive X-ray spectrometry (EDX)-mapping micrographs were obtained using a JEOL JEM-2100F microscope operating at 200 kV. *In situ* heating HAADF-STEM observation was performed by directly heating the sample in the column of JEOL JEM-2100F microscope with a double tilt heating holder (GATAN model 652). The pressure inside the column of this electron microscope was kept below 4 × 10^−5^ Pa. Pt particle size distribution histograms were obtained through measurement of at least 100 randomly selected nanoparticles. Scanning electron microscopy (SEM) and scanning transmission electron microscopy (STEM) images were obtained using a Hitachi S-4800 field-emission scanning electron microscope operating at 10 kV and 30 kV, respectively. The Pt loading of the sample was determined by inductively coupled plasma mass spectrometry (ICP-MS) over a ELAN 600 system. Nitrogen adsorption-desorption isotherms were acquired using a Quantachrome Autosorb-iQ analyzer at −196 °C. Before the measurement, degassing was conducted at 120 °C for 12 h. The Brunauer-Emmett-Teller (BET) specific surface area was calculated based on the adsorption isotherm. The pore size distribution was calculated from the adsorption branch using the Barrett-Joyner-Halenda (BJH) method. The total pore volume was estimated from the amount adsorbed at a relative pressure (P/P_0_) of 0.99. Pulse CO chemisorption was conducted on a Micromeritics AutoChem II 2910 instrument at 25 °C. Before the chemisorption, samples were heated to 200 °C for 1 h in the vacuum chamber to remove the adsorbed water, and then cooled down to room temperature and subjected to CO gas.

## Additional Information

**How to cite this article:** Liu, J. *et al*. Sintering-Resistant Nanoparticles in Wide-Mouthed Compartments for Sustained Catalytic Performance. *Sci. Rep.* **7**, 41773; doi: 10.1038/srep41773 (2017).

**Publisher's note:** Springer Nature remains neutral with regard to jurisdictional claims in published maps and institutional affiliations.

## Supplementary Material

Supporting Information

## References

[b1] Hansen T. W., Delariva A. T., Challa S. R. & Datye A. K. Sintering of catalytic nanoparticles: particle migration or Ostwald ripening? Acc. Chem. Res. 46, 1720–1730 (2013). 23634641 10.1021/ar3002427

[b2] Challa S. R. . Relating rates of catalyst sintering to the disappearance of individual nanoparticles during Ostwald ripening. J. Am. Chem. Soc. 133, 20672–20675 (2011). 22087502 10.1021/ja208324n

[b3] Simonsen S. B. . Ostwald ripening in a Pt/SiO_2_ model catalyst studied by *in situ* TEM. J. Catal. 281, 147–155 (2011).

[b4] Wettergren K. . High sintering resistance of size-selected platinum cluster catalysts by suppressed Ostwald ripening. Nano Lett. 14, 5803–5809 (2014). 25198035 10.1021/nl502686u

[b5] Cao A., Lu R. & Veser G. Stabilizing metal nanoparticles for heterogeneous catalysis. Phys. Chem. Chem. Phys. 12, 13499–13510 (2010). 20820585 10.1039/c0cp00729c

[b6] Behafarid F. & Cuenya B. R. Towards the understanding of sintering phenomena at the nanoscale: geometric and environmental effects. Top Catal. 56, 1532–1559 (2013).

[b7] Cheng L., Wang C., Feng L., Yang K. & Liu Z. Functional nanomaterials for phototherapies of cancer. Chem. Rev. 114, 10869–10939 (2014). 25260098 10.1021/cr400532z

[b8] Zhao Y., Shang L., Cheng Y. & Gu Z. Spherical colloidal photonic crystals. Acc. Chem. Res. 47, 3632–3642 (2014). 25393430 10.1021/ar500317s

[b9] Jing L. . Magnetically engineered semiconductor quantum dots as multimodal imaging probes. Adv. Mater. 26, 6367–6386 (2014). 25178258 10.1002/adma.201402296

[b10] Kim B. H., Hackett M. J., Park J. & Hyeon T. Synthesis, characterization, and application of ultrasmall nanoparticles. Chem. Mater. 26, 59–71 (2014).

[b11] Arnal P. M., Comotti M. & Schüth F. High-temperature-stable catalysts by hollow sphere encapsulation. Angew. Chem. Int. Ed. 45, 8224–8227 (2006). 10.1002/anie.200603507 17109458

[b12] Joo S. H. . Thermally stable Pt/mesoporous silica core-shell nanocatalysts for high-temperature reactions. Nat. Mater. 8, 126–131 (2009). 19029893 10.1038/nmat2329

[b13] Wu C. . A soft-templated method to synthesize sintering-resistant Au-mesoporous-silica core-shell nanocatalysts with sub-5 nm single-cores. Chem. Commun. 49, 3215–3217 (2013). 10.1039/c3cc39202c 23482935

[b14] Lu J. . Coking- and sintering-resistant palladium catalysts achieved through atomic layer deposition. Science 335, 1205–1208 (2012). 22403386 10.1126/science.1212906

[b15] Zhang T. . Unconventional route to encapsulated ultrasmall gold nanoparticles for high-temperature. ACS Nano 7, 7297–7304 (2014). 10.1021/nn502349k 24984223

[b16] Zhou H. . Thermally stable Pt/CeO_2_ hetero-nanocomposites with high catalytic activity. J. Am. Chem. Soc. 132, 4998–4999 (2010). 20329793 10.1021/ja101110m

[b17] Yoon K. . A highly reactive and sinter-resistant catalytic system based on platinum nanoparticles embedded in the inner surfaces of CeO_2_ hollow fibers. Angew. Chem. Int. Ed. 51, 9543–9546 (2012). 10.1002/anie.201203755 22930556

[b18] Xiao C. . High-temperature-stable and regenerable catalysts: platinum nanoparticles in aligned mesoporous silica wells. ChemSusChem 6, 1915–1922 (2013). 24039118 10.1002/cssc.201300524

[b19] Zhang N. & Xu Y. Aggregation- and leaching-resistant, reusable, and multifunctional Pd@CeO_2_ as a robust nanocatalyst achieved by a hollow core-shell strategy. Chem. Mater. 25, 1979–1988 (2013).

[b20] Shang L. . Graphene-supported ultrafine metal nanoparticles encapsulated by mesoporous silica: robust catalysts for oxidation and reduction reactions. Angew. Chem. Int. Ed. 53, 250–254 (2014). 10.1002/anie.201306863 24288240

[b21] Jiang H. . Au@ZIF-8: CO oxidation over gold nanoparticles deposited to metal-organic framework. J. Am. Chem. Soc. 131, 11302–11303 (2009). 19637919 10.1021/ja9047653

[b22] Witham C. A. . Converting homogeneous to heterogeneous in electrophilic catalysis using monodisperse metal nanoparticles. Nat. Chem. 2, 36–41 (2010). 21124378 10.1038/nchem.468

[b23] Gross E., Liu J. H. C., Toste F. D. & Somorjai G. A. Control of selectivity in heterogeneous catalysis by tuning nanoparticle properties and residence time. Nat. Chem. 4, 947–952 (2012). 23089871 10.1038/nchem.1465

[b24] Prieto G., Zečević J., Friedrich H., de Jong K. P. & de Jongh P. E. Towards stable catalysts by controlling collective properties of supported metal nanoparticles. Nat. Mater. 12, 34–39 (2013). 23142841 10.1038/nmat3471

[b25] Munnik P., de Jongh P. E. & de Jong K. P. Control and impact of the nanoscale distribution of supported cobalt particles used in Fischer-Tropsch catalysis. J. Am. Chem. Soc. 136, 7333–7340 (2014). 24801898 10.1021/ja500436y

[b26] Crooks R. M., Zhao M., Sun L., Chechik V. & Yeung L. K. Dendrimer-encapsulated metal nanoparticles: synthesis, characterization, and applications to catalysis. Acc. Chem. Res. 34, 181–190 (2001). 11263876 10.1021/ar000110a

[b27] Ji Q. . Flake-shell capsules: adjustable inorganic structures. Small 8, 2345–2349 (2012). 22566345 10.1002/smll.201200317

[b28] Lang H., May R. A., Iversen B. L. & Chandler B. D. Dendrimer-encapsulated nanoparticle precursors to supported platinum catalysts. J. Am. Chem. Soc. 125, 14832–14836 (2003). 14640659 10.1021/ja0364120

[b29] Astruc D., Boisselier E. & Ornelas C. Dendrimers designed for functions: from physical, photophysical, and supramolecular properties to applications in sensing, catalysis, molecular electronics, photonics, and nanomedicine. Chem. Rev. 110, 1857–1959 (2010). 20356105 10.1021/cr900327d

[b30] Ding S. . Biomolecule-assisted route to prepare titania mesoporous hollow structures. Chem. Eur. J. 17, 11535–11541 (2011). 21882272 10.1002/chem.201101314

[b31] Sun C. . Mesoscale organization of nearly monodisperse flowerlike ceria microspheres. J. Phys. Chem. B 110, 13445–13452 (2006). 16821869 10.1021/jp062179r

[b32] Li Z. . Fabrication of hierarchically assembled microspheres consisting of nanoporous ZnO nanosheets for high-efficiency dye-sensitized solar cells. J. Mater. Chem. 22, 14341–14345 (2012).

[b33] Zhong L. . Self-assembled 3D flowerlike iron oxide nanostructures and their application in water treatment. Adv. Mater. 18, 2426–2431 (2006).

[b34] Fei J. . Controlled preparation of MnO_2_ hierarchical hollow nanostructures and their application in water treatment. Adv. Mater. 20, 452–456 (2008).

[b35] Purushothaman K. K., Babu I. M., Sethuraman B. & Muralidharan G. Nanosheet-assembled NiO microstructures for high-performances. ACS Appl. Mater. Interfaces 5, 10767–10773 (2013). 24124992 10.1021/am402869p

[b36] Wang X. . Synthesis and lithium storage properties of Co_3_O_4_ nanosheet-assembled multishelled hollow spheres. Adv. Funct. Mater. 20, 1680–1686 (2010).

[b37] Bai S. . Synthesis mechanism and gas-sensing application of nanosheet tungsten oxide microspheres. J. Mater. Chem. A 2, 7927–7934 (2014).

[b38] Nie L., Meng A., Yu J. & Jaroniec M. Hierarchically macro-mesoporous Pt/γ-Al_2_O_3_ composite microspheres for efficient formaldehyde oxidation at room temperature. Sci. Rep. 3, 3215 (2013). 24225532 10.1038/srep03215 PMC3827609

[b39] Li W. . Hydrothermal etching assisted crystallization: a facile route to functional yolk-shell titanate microspheres with ultrathin nanosheets-assembled double shells. J. Am. Chem. Soc. 133, 15830–15833 (2011). 21905658 10.1021/ja2055287

[b40] Pan A., Wu H., Zhang L. & Lou X. W. D. Nanosheet-assembled hollow microflowers with excellent lithium storage properties. Energy Environ. Sci. 6, 1476–1479 (2013).

[b41] Olah G. A., Goeppert A., Czaun M. & Prakash G. K. S. Bi-reforming of methane from any source with steam and carbon dioxide exclusively to Metgas (CO-2H_2_) for methanol and hydrocarbon synthesis. J. Am. Chem. Soc. 135, 648–650 (2013). 23256664 10.1021/ja311796n

[b42] Li S. & Gong J. Strategies for improving the performance and stability of Ni-based catalysts for reforming reactions. Chem. Soc. Rev. 43, 7245–7256 (2014). 25182070 10.1039/c4cs00223g

[b43] Zhao D., Huo Q., Feng J., Chmelka B. F. & Stucky G. D. Nonionic triblock and star diblock dopolymer and oligomeric surfactant syntheses of highly ordered, hydrothermally stable, mesoporous silica structures. J. Am. Chem. Soc. 120, 6024–6036 (1998).

[b44] Rioux R. M., Song H., Hoefelmeyer J. D., Yang P. & Somorjai G. A. High-surface-area catalyst design: synthesis, characterization, and reaction studies of platinum nanoparticles in mesoporous SBA-15 silica. J. Phys. Chem. B 109, 2192–2202 (2005). 16851211 10.1021/jp048867x

