# Supplementary material for: Sintering-Resistant Nanoparticles in Wide-Mouthed Compartments for Sustained Catalytic Performance
Source: Sci Rep. 2017 Feb 3;7:41773. doi: 10.1038/srep41773 (PMC5290533; doi:10.1038/srep41773)
Supplement: Supporting Information [file srep41773-s1.pdf]

Supporting Information

Sintering-Resistant Nanoparticles in Wide-Mouthed Compartments for Sustained Catalytic Performance

Jia Liu,[a] Qingmin Ji,[a] Tsubasa Imai,[b] Katsuhiko Ariga,*[a] and Hideki Abe*[b]

[a] World Premier International (WPI) Research Center for Materials Nanoarchitectonics (MANA), National Institute for Materials Science (NIMS), Namiki 1-1, Tsukuba, Ibaraki 305-0044, Japan

[b] Environment and Energy Materials Division, National Institute for Materials Science (NIMS), Namiki 1-1, Tsukuba, Ibaraki 305-0044, Japan

Contents

Supplementary Scheme S1: Preparation procedures for different samples and their corresponding Pt particle size……..……………………………………………………3
Supplementary Table S1: Textural parameters for the samples……………………………4
Supplementary Table S2: Pt loading weights for the samples………………………………4
Supplementary Figure S1: TEM image of PtDEN…………………………………………5
Supplementary Figure S2: STEM and HAADF-STEM images of CMPT……...………………5
Supplementary Figure S3: SEM and TEM images; N_2 sorption isotherms; pore size distribution of SBA15…………………………………………………………………………………6
Supplementary Figure S4: SEM image of the silica nanospheres……………………………6
Supplementary Figure S5: Particle size distribution of Pt for PtDEN/CMPT, PtDEN/SBA15 and PtDEN/NS...7
Supplementary Figure S6: SEM, HAADF-STEM and TEM images of Pt/CMPT……………..8
Supplementary Figure S7: HAADF-STEM images and the corresponding elemental mapping images of Pt/CMPT and Pt/NS …………………...…………………………………………9
Supplementary FigureS8: HRTEM images of Pt/CMPT, Pt/SBA15 and Pt/NS………………..9
Supplementary Figure S9: TEM images of Pt/CMPT samples with different Pt loading……..10
Supplementary Figure S10: Pt particle size distribution for the Pt/CMPT samples with different Pt loading…..11
Supplementary Figure S11: TEM, HAADF-STEM images of Pt/CMPT, Pt/SBA15 and Pt/NS after CO oxidation……………………………………………………………………………..12
Supplementary Figure S12: Pt particle size distribution for the Pt/CMPT, Pt/SBA15 and Pt/NS after CO oxidation ……………………………………………………………………………13

Supplementary Scheme S1. Preparation procedures for different samples and their corresponding Pt particle size.

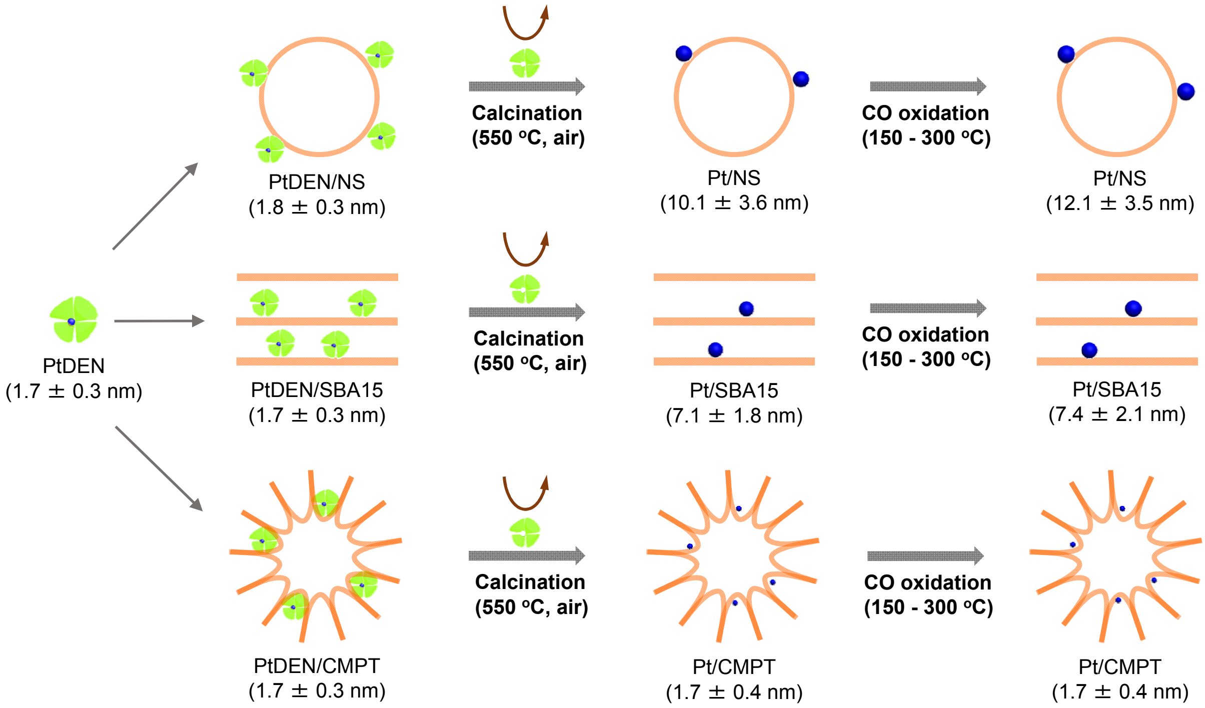

Supplementary Table S1. Textural parameters of different samples.

Samples	BET surface area (m^2 g^{-1})	Pore volume (cm^3 g^{-1})	Pore size (nm)
CMPT[a]	695	1.3	—
Pt/CMPT	497	0.9	—
Pt/CMPT[b]	511	1.0	—
NS	58	—	—
SBA15	644	1.2	8.1
Pt/SBA15	628	1.2	8.1
Pt/SBA15[b]	562	1.1	8.1

[a] Data from previous publication (*Small* **8,** 2345-2349 (2012)).

[b] After CO oxidation in the temperature range of 150 - 300 °C.

Supplementary Table S2. Pt loading for different samples as determined by inductively coupled plasma mass spectrometry (ICP-MS).

**Samples**	**Pt loading (wt%)**[a]
Pt/CMPT	0.075[b]
Pt/SBA15	0.060
Pt/NS	0.065

[a] The theoretical Pt loading is 0.070 wt%.

[b] For this Pt loading, the ratio of compartments offered by the CMPT support to Pt nanoparticles was roughly estimated to be higher than 7. In specific, the average diameter of the CMPT particles is around 500 nm, thus the volume of a CMPT particle could be calculated at 6.54×10^{-20} m^3, and its corresponding weight is 1.03×10^{-15} g on account of a measured density of 1.58×10^{-2} g cm^{-3}. Therefore 1 g of CMPT should contain about 9.71×10^{14} particles. The number of compartments in one CMPT particle is very hard to determine but obviously it should be higher than 100. Thus, one can speculate that 1 g of CMPT could offer more than 9.71×10^{16} compartments. On the other hand, the average diameter of Pt nanoparticles is 1.7 nm and the density of Pt is 21.45 g cm^{-3}, based on which the average weight of one Pt nanoparticle was calculated at 5.52×10^{-20} g. When the Pt loading was controlled at 0.075 wt%, 1 g of the Pt/CMPT sample should contain about 1.36×10^{16} Pt nanoparticles, and finally the ratio of compartments to Pt nanoparticles was calculated to be higher than 7.14.

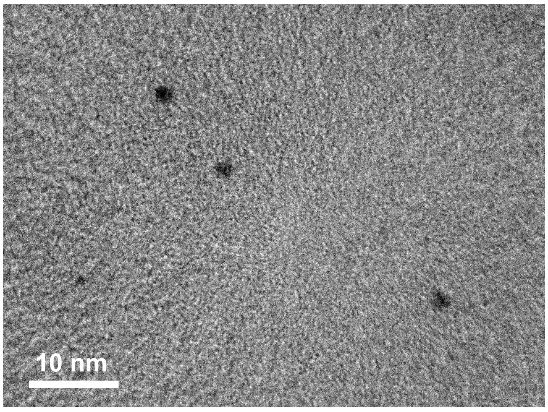

Supplementary Figure S1. TEM image of Pt dendrimer-encapsulated nanoparticles (PtDEN). Six-generation, hydroxyl-terminated poly(amidoamine)(PAMAM) dendrimer was used as the capping agent for the preparation of PtDEN.

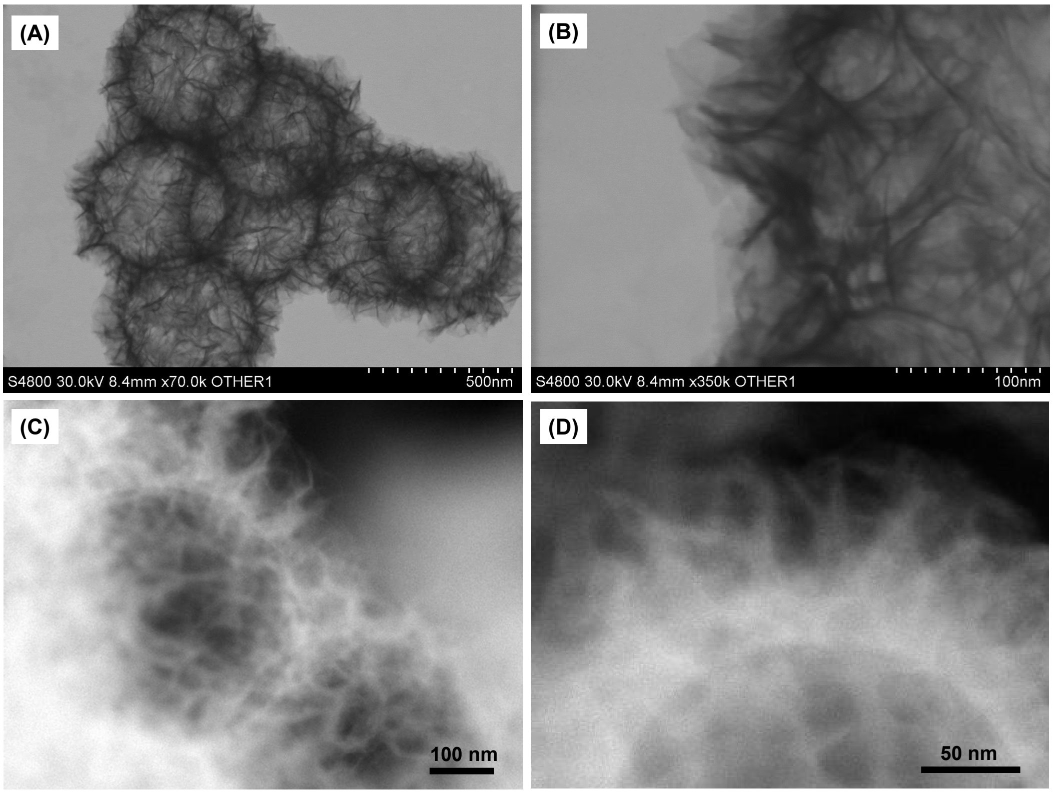

Supplementary Figure S2. (A, B) STEM and (C, D) HAADF-STEM images of the compartment-rich silicas (CMPT) constituted by the three-dimensional assembly of tremendous silica nanosheets with thickness of about 5 nm.

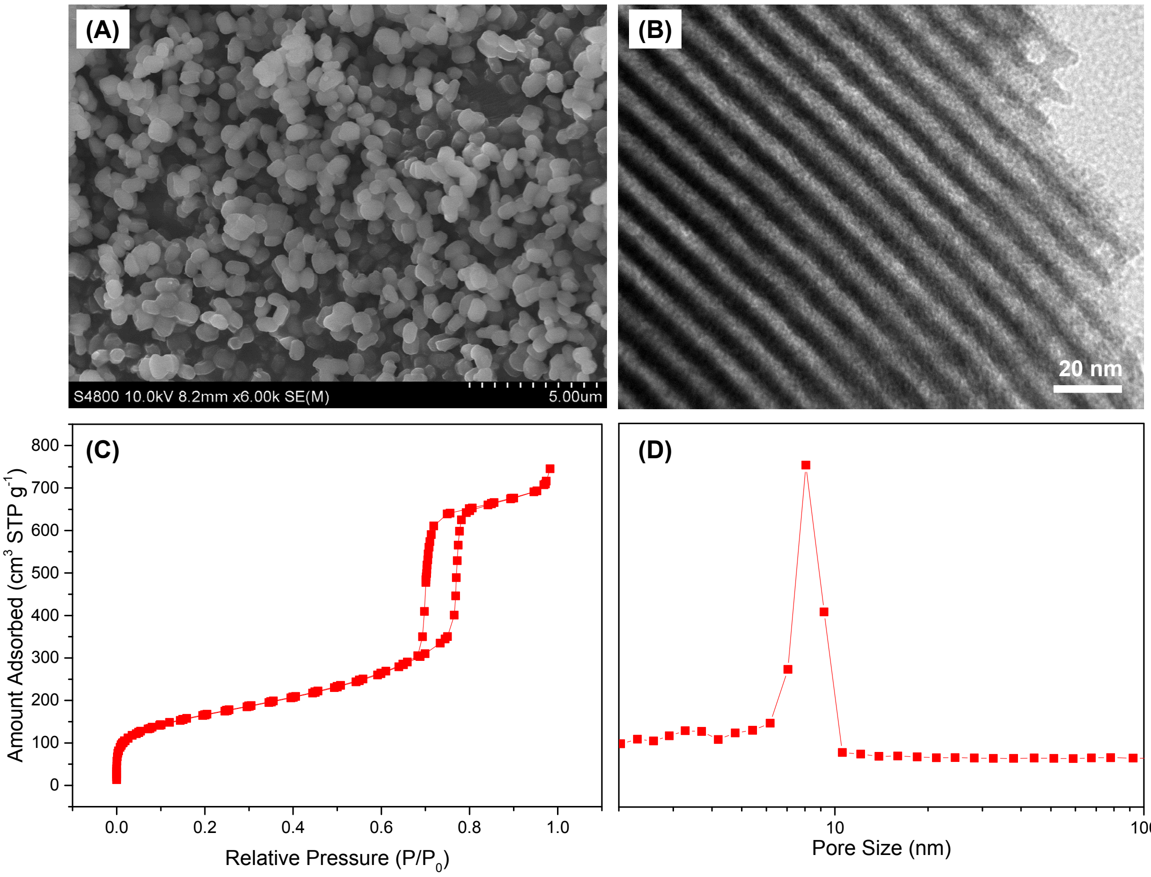

Supplementary Figure S3. (A) SEM image, (B) TEM image, (C) nitrogen adsorption-desorption isotherms and (D) pore size distribution of mesoporous silica SBA15.

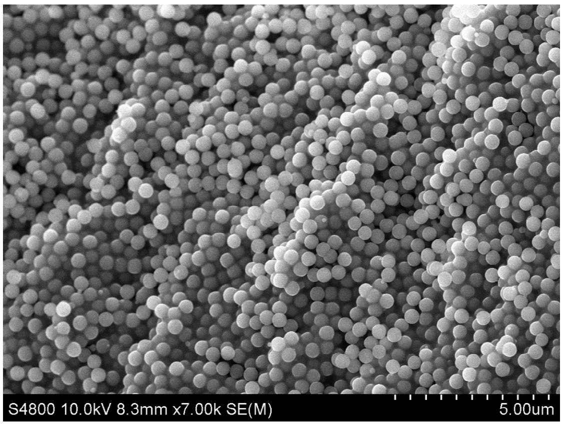

Supplementary Figure S4. SEM image of silica nanospheres (NS). The particle diameter of NS is in the range of 500-550 nm, similar to that of CMPT.

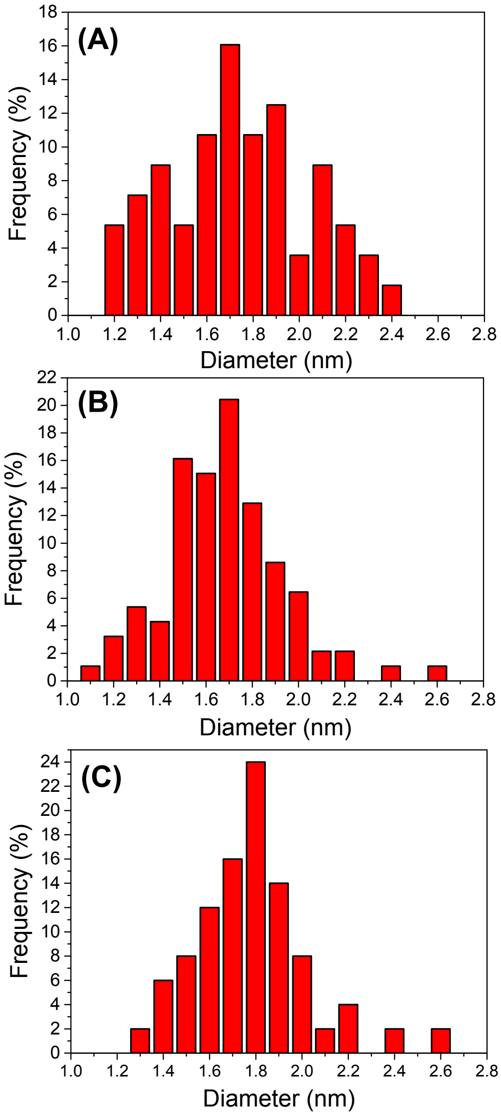

Supplementary Figure S5. Pt particle size distribution histogram of (A) PtDEN/CMPT, (B) PtDEN/SBA15 and (C) PtDEN/NS.

The average Pt particle size for PtDEN/CMPT, PtDEN/SBA15 and PtDEN/NS was 1.7 ± 0.3, 1.7 ± 0.3 and 1.8 ± 0.3 nm, respectively. The slightly larger Pt particle size for PtDEN/NS was probably caused by the measurement error relating to STEM analysis. Unlike the other two samples, TEM was not adaptable to PtDEN/NS due to the insufficient contrast between the small Pt and large silica particles.

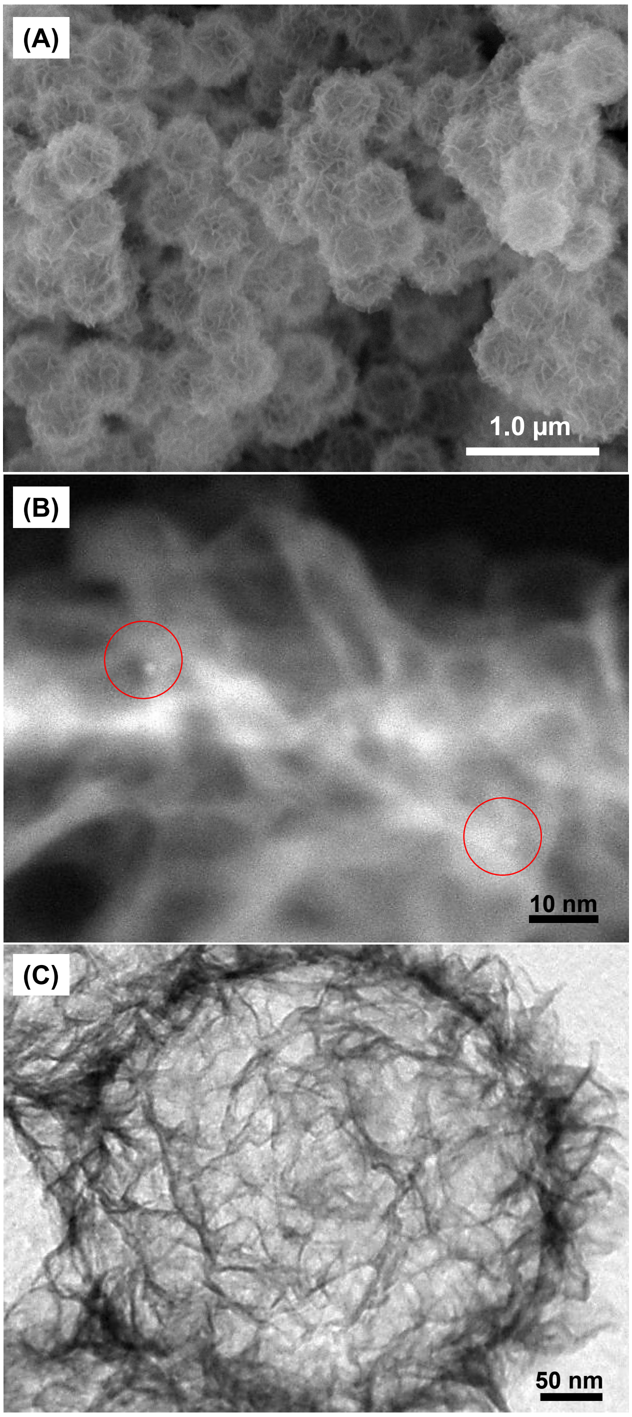

Supplementary Figure S6. (A) SEM, (B) HAADF-STEM and (C) TEM images of Pt/CMPT.

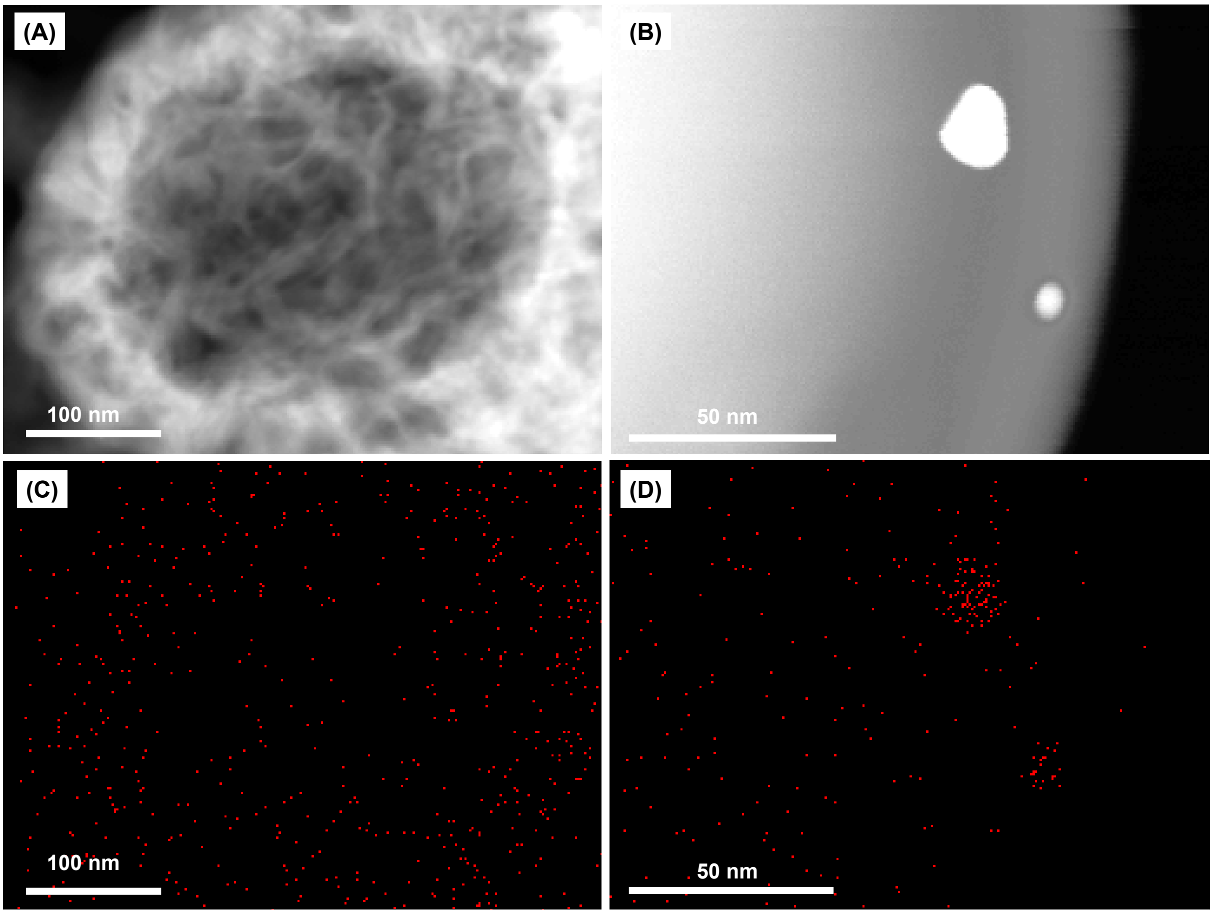

Supplementary Figure S7. (A, B) HAADF-STEM images and (C, D) the corresponding mapping results of element Pt for (A, C) Pt/CMPT and (B, D) Pt/NS.

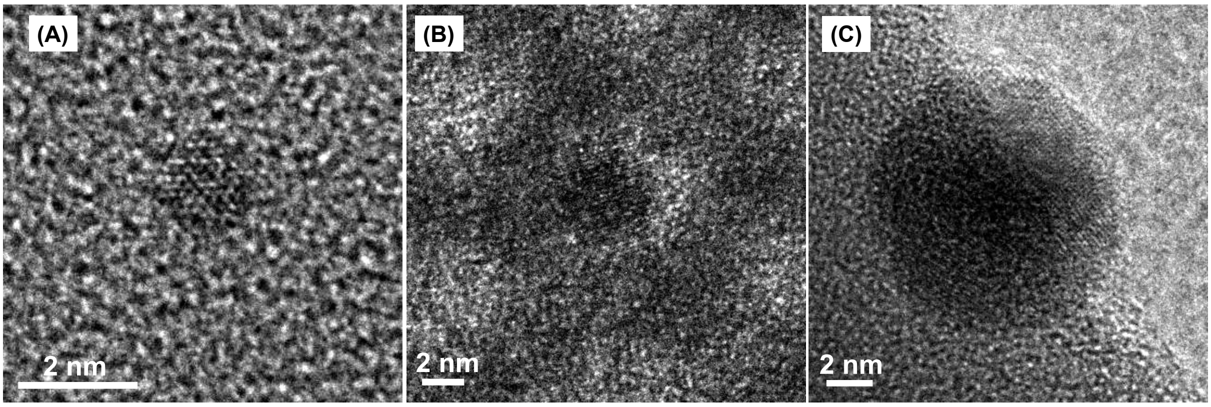

Supplementary Figure S8. High resolution TEM images of Pt nanoparticles for (A) Pt/CMPT, (B) Pt/SBA15 and (C) Pt/NS.

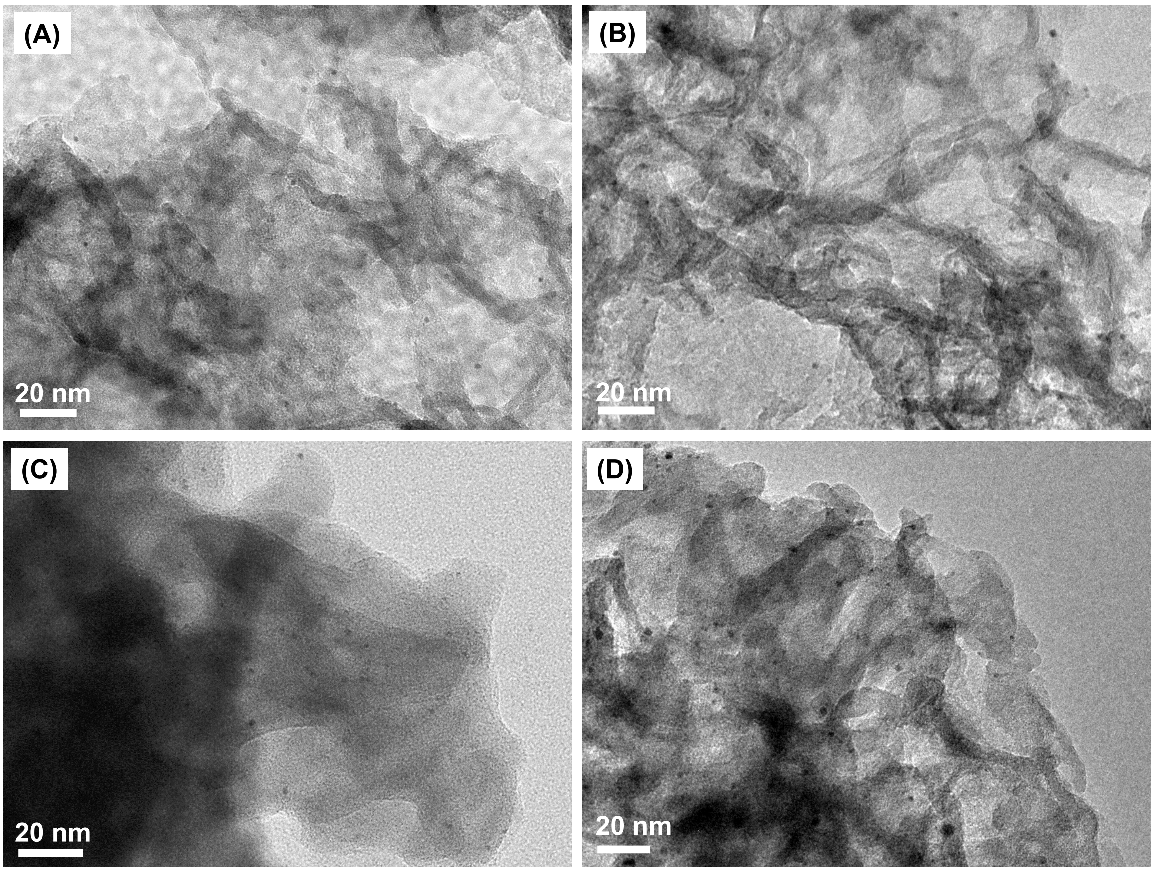

Supplementary Figure S9. TEM images of Pt/CMPT samples with the Pt loading varied at (A) 0.21 wt%, (B) 0.35 wt%, (C) 0.49 wt% and (D) 0.84 wt%, respectively.

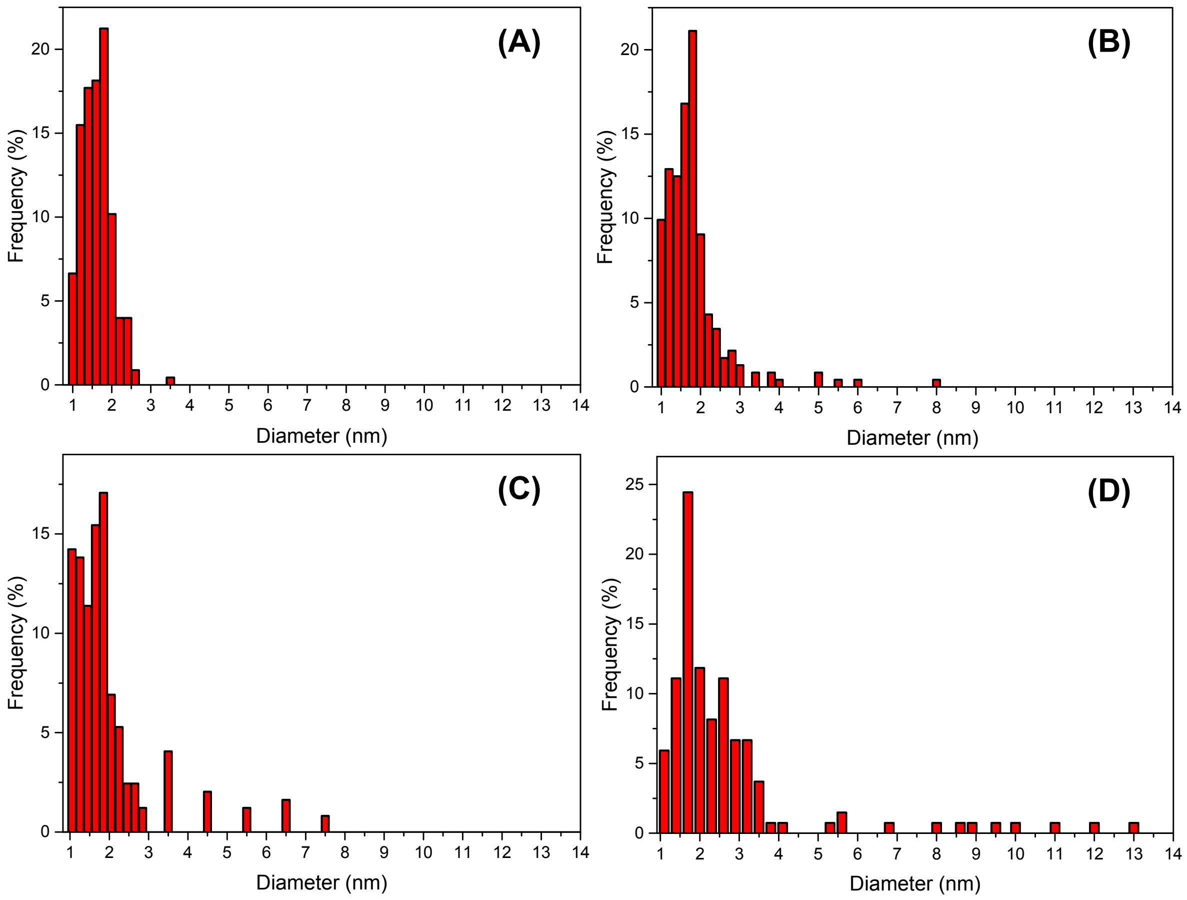

Supplementary Figure S10. Pt particle size distribution histogram for Pt/CMPT samples with the Pt loading varied at (A) 0.21 wt%, (B) 0.35 wt%, (C) 0.49 wt% and (D) 0.84 wt%, respectively.

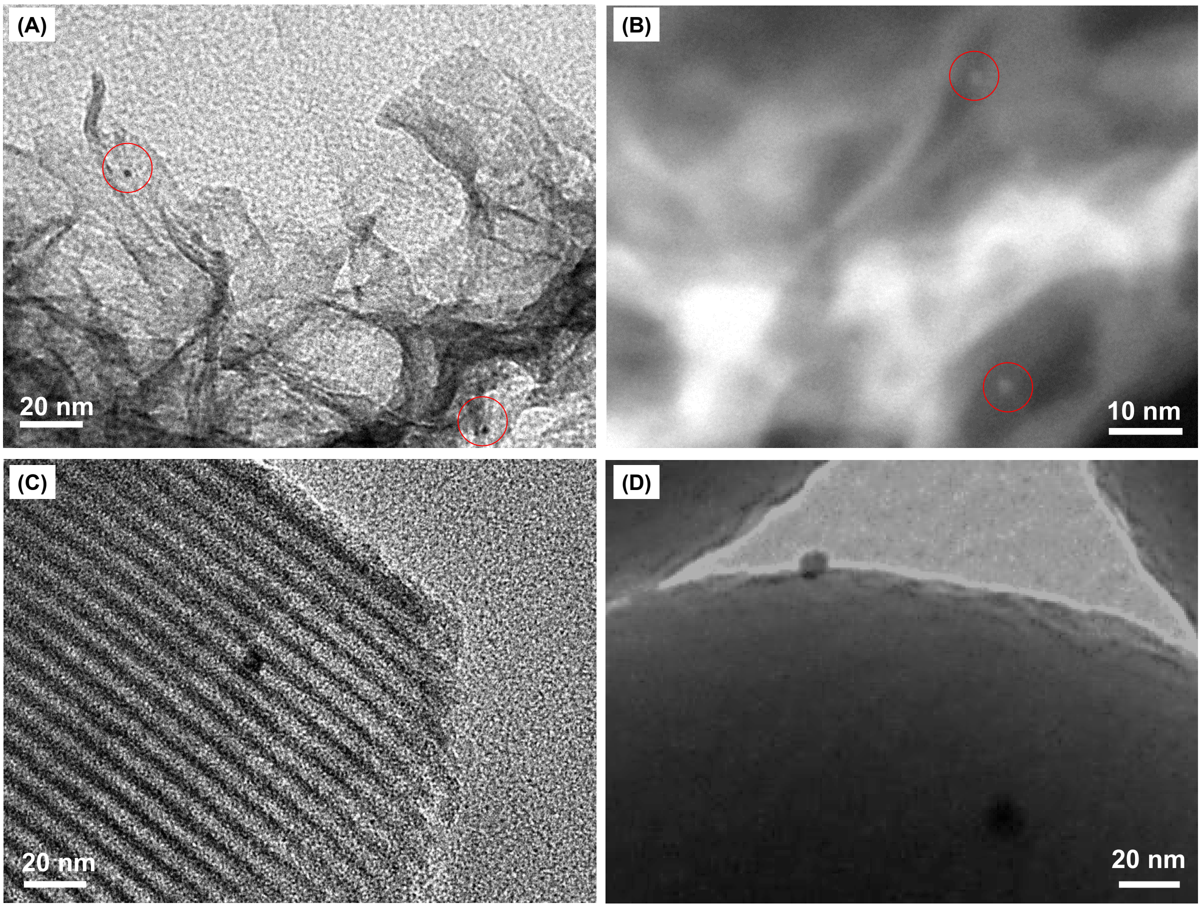

Supplementary Figure S11. (A, C, D) TEM images and (B) HAADF-STEM image of (A, B) Pt/CMPT, (C) Pt/SBA15 and (D) Pt/NS after CO oxidation in the temperature range of 150 - 300 ^{o}C.

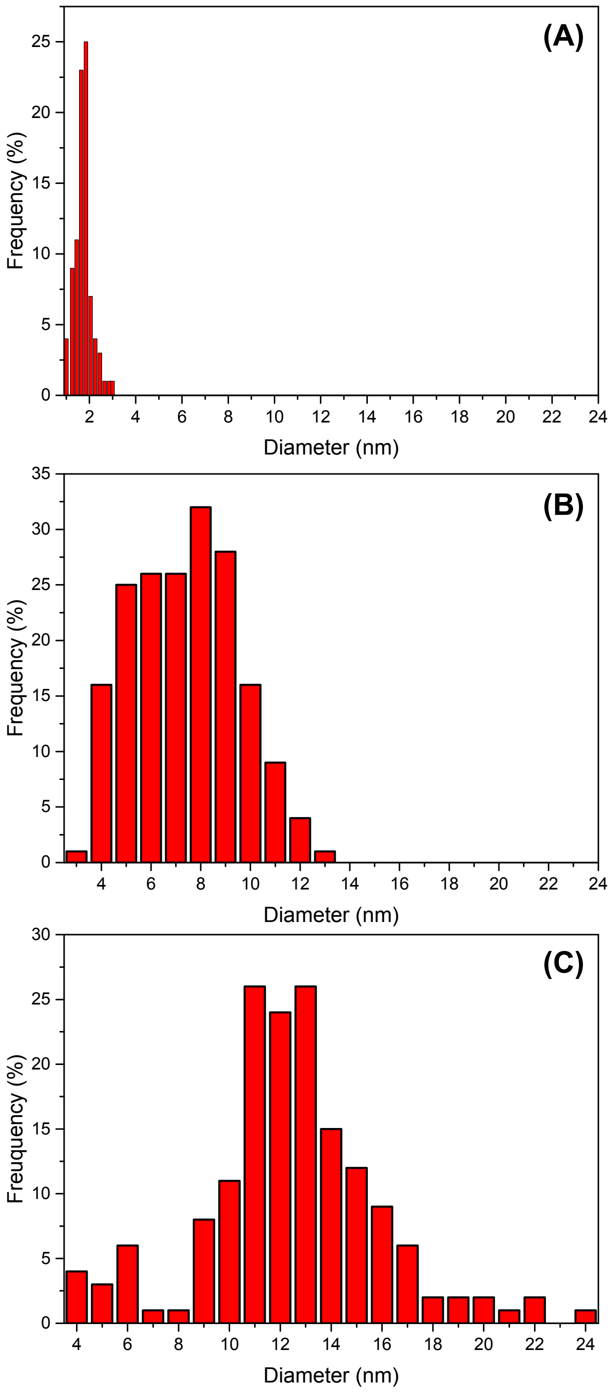

Supplementary Figure S12. Pt Particle size distribution histogram for (A) Pt/CMPT, (B) Pt/SBA15 and (C) Pt/NS after CO oxidation in the temperature range of 150 - 300 ^{o}C.
